# Aptamer-Conjugated Superparamagnetic Ferroarabinogalactan Nanoparticles for Targeted Magnetodynamic Therapy of Cancer

**DOI:** 10.3390/cancers12010216

**Published:** 2020-01-15

**Authors:** Olga S. Kolovskaya, Tatiana N. Zamay, Galina S. Zamay, Vasily A. Babkin, Elena N. Medvedeva, Nadezhda A. Neverova, Andrey K. Kirichenko, Sergey S. Zamay, Ivan N. Lapin, Evgeny V. Morozov, Alexey E. Sokolov, Andrey A. Narodov, Dmitri G. Fedorov, Felix N. Tomilin, Vladimir N. Zabluda, Yulia Alekhina, Kirill A. Lukyanenko, Yury E. Glazyrin, Valery A. Svetlichnyi, Maxim V. Berezovski, Anna S. Kichkailo

**Affiliations:** 1 Federal Research Center “Krasnoyarsk Science Center of the Siberian Branch of the Russian Academy of Science”, 660036 Krasnoyarsk, Russia; olga.kolovskaya@gmail.com (O.S.K.); galina.zamay@gmail.com (G.S.Z.); sergey-zamay@yandex.ru (S.S.Z.); felixnt@gmail.com (F.N.T.); kirill.lukyanenko@gmail.com (K.A.L.); yury.glazyrin@gmail.com (Y.E.G.); 2 Laboratory for Biomolecular and Medical Technologies, Faculty of Medicine, Krasnoyarsk State Medical University named after prof. V.F. Voino-Yasenecki, 660022 Krasnoyarsk, Russia; tzamay@yandex.ru (T.N.Z.); krasak.07@mail.ru (A.K.K.); narodov_a@mail.ru (A.A.N.); 3 Irkutsk Institute of Chemistry named after A.E. Favorsky, the Siberian Branch of the Russian Academy of Sciences, 664033 Irkutsk, Russia; babkin@irioch.irk.ru (V.A.B.); l.medwedewa2009@yandex.ru (E.N.M.); nadya_neverova@irioch.irk.ru (N.A.N.); 4 L.V. Kirensky Institute of Physics SB RAS—The Branch of Federal Research Center “Krasnoyarsk Science Center of the Siberian Branch of the Russian Academy of Sciences”, 660036 Krasnoyarsk, Russia; morozov_if@mail.ru (E.V.M.); alexeys@iph.krasn.ru (A.E.S.); zvn@iph.krasn.ru (V.N.Z.); 5 Laboratory of Advanced Materials and Technology, Tomsk State University, 634050 Tomsk, Russia; 201kiop@mail.ru (I.N.L.); v_svetlichnyi@bk.ru (V.A.S.); 6 Institute of Chemistry and Chemical Technology SB RAS—The Branch of Federal Research Center “Krasnoyarsk Science Center of the Siberian Branch of the Russian Academy of Sciences”, 660036 Krasnoyarsk, Russia; 7 School of Engineering Physics and Radio Electronics, Siberian Federal University, 660041 Krasnoyarsk, Russia; 8 Research Center for Computational Design of Advanced Functional Materials (CD-FMat), National Institute of Advanced Industrial Science and Technology (AIST), Tsukuba 305-8568, Japan; d.g.fedorov@aist.go.jp; 9 School of Non-Ferrous Metals and Materials Science, Siberian Federal University, 660041 Krasnoyarsk, Russia; 10 Faculty of Physics, Department of Magnetism, Lomonosov Moscow State University, 119991 Moscow, Russia; ya.alekhina@physics.msu.ru; 11 School of Fundamental Biology and Biotechnology, Siberian Federal University, 660041 Krasnoyarsk, Russia; 12 Department of Chemistry and Biomolecular Sciences, University of Ottawa, Ottawa, ON K1N 6N5, Canada

**Keywords:** aptamers, arabinogalactan, superparamagnetic ferroarabinogalactans, drug delivery, magnetodynamic therapy, magnetically induced cell disruption, magnetic resonance imaging

## Abstract

Nanotechnologies involving physical methods of tumor destruction using functional oligonucleotides are promising for targeted cancer therapy. Our study presents magnetodynamic therapy for selective elimination of tumor cells in vivo using DNA aptamer-functionalized magnetic nanoparticles exposed to a low frequency alternating magnetic field. We developed an enhanced targeting approach of cancer cells with aptamers and arabinogalactan. Aptamers to fibronectin (AS-14) and heat shock cognate 71 kDa protein (AS-42) facilitated the delivery of the nanoparticles to Ehrlich carcinoma cells, and arabinogalactan (AG) promoted internalization through asialoglycoprotein receptors. Specific delivery of the aptamer-modified FeAG nanoparticles to the tumor site was confirmed by magnetic resonance imaging (MRI). After the following treatment with a low frequency alternating magnetic field, AS-FeAG caused cancer cell death in vitro and tumor reduction in vivo. Histological analyses showed mechanical disruption of tumor tissues, total necrosis, cell lysis, and disruption of the extracellular matrix. The enhanced targeted magnetic theranostics with the aptamer conjugated superparamagnetic ferroarabinogalactans opens up a new venue for making biocompatible contrasting agents for MRI imaging and performing non-invasive anti-cancer therapies with a deep penetrated magnetic field.

## 1. Introduction

Current advances in the synthesis of aptamer conjugates with various drugs, biopolymers, and even nanoparticles enable the number of research studies in biomedicine to be increased. Big interest in the treatment of cancers is in the application of aptamer conjugates with nanostructures due to their high specificity for cancer cells and low toxicity for the whole organism [1,2]. Traditional small molecule antitumor drugs are highly toxic to normal cells. Magnetic nanoparticles are becoming important for targeted and low invasive cancer therapy [1,3,4]. To date, several ferromagnetic and superparamagnetic nanoparticles have already been applied for medical purposes [5,6,7]. Superparamagnetic particles are preferable to ferromagnetic because, in the absence of a magnetic field, they have no magnetic moment, and they act only when the magnetic field is applied. Such particles have been used for magnetic resonance imaging (MRI), hyperthermia induction, and mechanical destruction of cells. Particular attention should be paid to iron-containing ferroarabinogalactans (FeAGs) in which iron is stabilized by arabinogalactan (AG), a plant polysaccharide isolated from larch wood. Iron in these FeAGs is in the form of hydrated iron oxide with the general formula Fe_3_O_4_ × nH_2_O, an analog of minerals of magnetite–maghemite or ferrihydrites [8,9]. The magnetic properties of iron derivatives of AG have been described before [10]. The benefits of FeAG are low toxicity, along with antioxidant, immunomodulating, and detoxification properties. An excess of AG promotes the excretion of tumor decay products.

For improving targeted delivery, the nanoparticles could be modified with specific ligands-antibodies or nucleic acid aptamers. Production of aptamers is a hundred times cheaper than monoclonal antibodies. Nucleic acid aptamers have already been demonstrated as having enormous potential as agents for molecular recognition [11]. Aptamers are ideal candidates for therapy as delivery agents due to high selectivity and low immunogenicity [11,12]. They are selected through an in vitro evolution process in a few days to various targets, such as small molecule viruses, bacteria, proteins, live cells, tissues [11,12,13,14,15,16,17,18]. Aptamers are considered to be a synthetic chemical product, rather than biological because they are chemically synthesized in high purity by an automated procedure. Functionalization of nanoparticles with the aptamers improves their biocompatibility, colloidal stability, and increases the circulation time in vivo [19,20,21].

Here we show the production of oligonucleotide aptamer conjugates with superparamagnetic nanoparticles synthesized in a polymer matrix of AG, as well as a novel application for targeted cancer therapy in a low frequency alternating magnetic field (LFAMF).

## 2. Results and Discussion

### 2.1. Production of Different Compositions of Superparamagnetic FeAG

Wet chemical preparation for nanoparticle synthesis is preferable due to scalability and fine control over the final particle structural properties [22]. The most common strategy for producing superparamagnetic particles of 10–20 nm in diameter involves aqueous precipitation of iron salts in situ [22,23,24]. Many polymeric materials, such as dextran, carboxymethylated dextran, carboxydextran, starch, PEG, AG, glycosaminoglycan, organic siloxane, and sulfonated styrene have been proposed for coating iron oxide nanoparticles [25,26].

**Figure 1:**
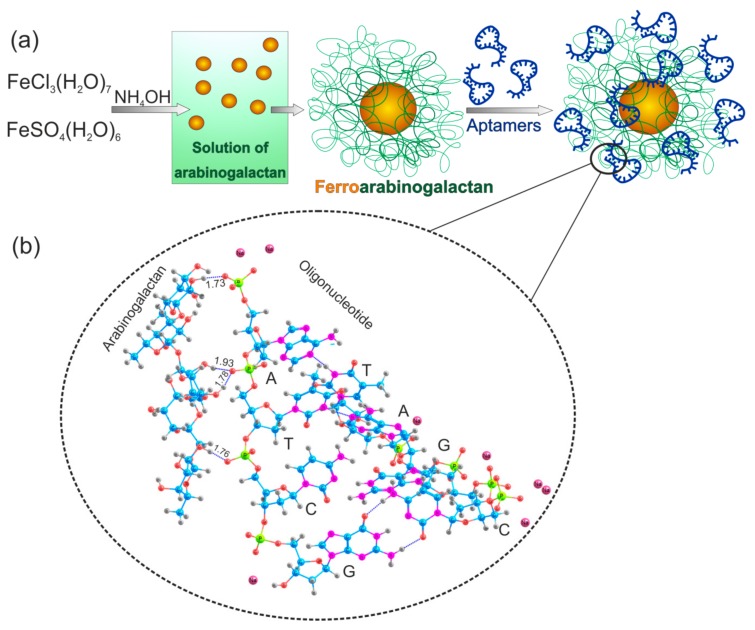
Synthesis of aptamer-conjugated ferroarabinogalactan nanoparticles (AS-FeAGs). (**a**) Schematic representation of the synthesis of ferroarabinogalactan nanoparticles and their conjugation with aptamers. (**b**) A part of the aptamer-arabinogalactan complex. C—Cytosine, G—Guanine, A—Adenine, and T—Thymine. Non-covalent hydrogen bonds between atoms are shown as blue dashed lines. Hydrogen—gray, oxygen—red, carbon—blue, nitrogen—purple, phosphorus—yellow, sodium—pink.

In this work, nano dispersed magnetite was encapsulated in the biopolymer matrix of natural polysaccharide AG (Figure 1a). For the preparation of iron-containing derivatives, the reaction of iron salts with ammonia in an aqueous solution of AG was used. The resulting ultra-dispersed polymer particles of the hydrated iron oxide have high surface energy due to their small size. Polysaccharide AG acts as a matrix for nanoparticle formation. This leads to the adsorption of AG on the particles. AG stabilizes the nanoparticles and prevents their aggregation. The reaction conditions and yields of the synthesized ferroarabinogalactan (FeAG) from full-length AG (FeAG, 16,645 Da) or fragmented AG (FrFeAG, 8380 Da) with different iron content are shown in Table 1.

### 2.2. Magnetic Characteristics of FeAG

**Figure 2:**
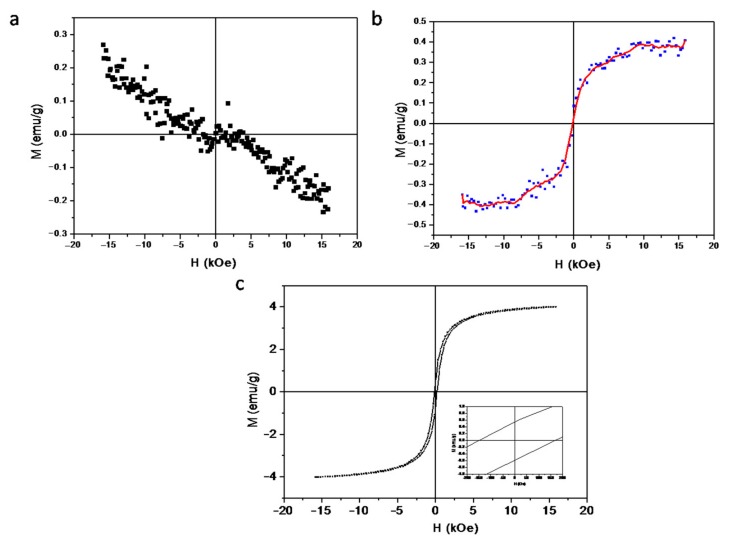
Magnetic properties of ferroarabinogalactan. (**a**) Pure arabinogalactan does not have a magnetic moment. (**b**) Subtraction of a diamagnetic curve of solvent from a curve of ferroarabinogalactan solution. (**c**) The magnetization of superparamagnetic nanodispersed ferroarabinogalactan.

Pure arabinogalactan does not have any magnetic properties and is diamagnetic (Figure 2a). FeAG magnetization in solution has a curve typical for a superparamagnetic compound (Figure 2b). The magnetization of ferroarabinogalactan powder demonstrates that FeAG has magnetic properties due to the "embedded" nano dispersed iron oxides, presumably maghemite (Fe_2_O_3_), and exhibits ferromagnetic or ferrimagnetic behaviour (Figure 2c). The saturation field of FeAG nanoparticles lies in the region of 3 kOe, and their coercivity is about 150 Oe. The saturation magnetization is 4 emu/g (Figure 2c). Therefore, LFAMF about 100 Oe is not enough to reverse ferroarabinogalactan magnetization but is enough to reorient nanoparticles in solution.

### 2.3. Conjugation of FeAG with Aptamers

Aptamers can be applied as a carrier and bring nanoparticles to cancer cells to improve their accumulation in a tumor [27,28,29]. For selective targeting, we used two aptamers (AS-14 and AS-42) with the specific binding to mouse Ehrlich carcinoma. These aptamers were selected and described in our previous study [30,31]. AS-14 binds to post-translationally modified fibronectin of extracellular matrix of cancer cells where threonines were acetylated at the position 1029 and phosphorylated at the position 1033. AS-42 binds to heat shock cognate 71 kDa (Hsc70) protein.

Conjugation with the aptamers of 500 nM was carried out by incubating FeAG in Dulbecco’s phosphate buffer saline (DPBS) buffer with calcium (II) and magnesium (II) at room temperature for 30 min.AG has a branched structure comprised of a backbone of 1,3-linked galactopyranose connected by 1,3-glycosidic linkages [32].

**Figure 3:**
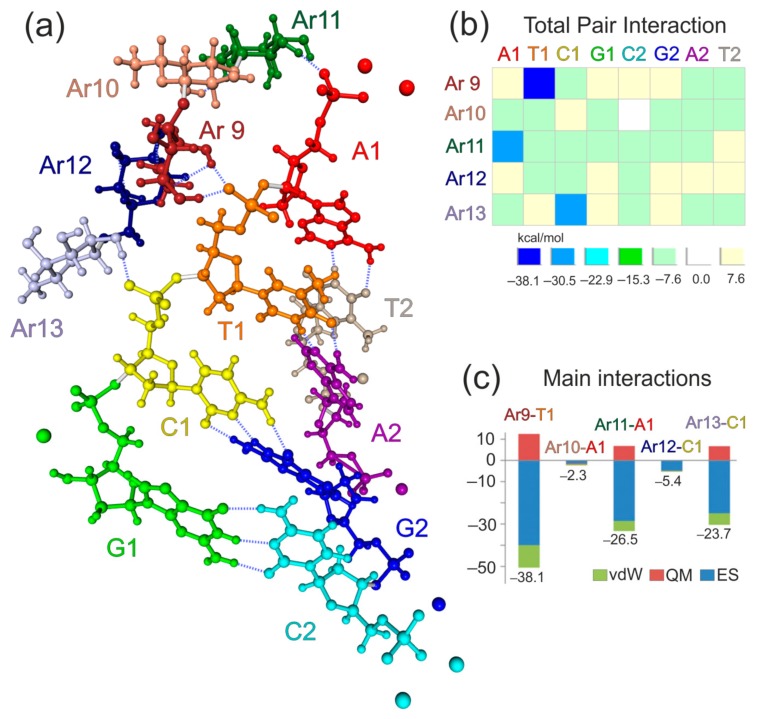
The molecular structure and results of the fragment molecular orbital method (FMO) applied to the aptamer-arabinogalactan complex. (**a**) Fragments of arabinogalactan and aptamer AS-14 are shown in different colours. (**b**) Total pair interactions between arabinogalactan (Ar9–Ar13) and aptamer fragments (A1, T1, C1, G1, A2, T2, C2, G2,). All values are in kcal/mol. (**c**) Main interactions between arabinogalactan (Ar9–Ar13) and aptamer fragments (T1, A1, C1). The total values are shown below each bar. All values are in kcal/mol. Each pair interaction is divided into electrostatic (ES), quantum-mechanical (QM), and van-der-Waals (vdW) components.

Computer simulations of a complex of AG with an aptamer were performed using high-level quantum-mechanical calculations, as described in detail in the Methods section. It was found in simulations that nucleotides of a DNA aptamer interact with monosaccharides of AG, forming a large number of hydrogen bonds (with lengths of 1.734 to 1.927 Å). Depending on a conformation, the monosaccharide can form one or two hydrogen bonds with oxygens in a phosphate group of a nucleotide. Figure 3a shows that hydrogen bonds are formed between A1 and Ar11 (one bond), T1 and Ar9 (two bonds), C1, and Ar13 (one bond). The quantitative picture was obtained using the pair interaction energy decomposition analysis (PIEDA). PIEDA components reveal characteristic signatures [33]. It confirmed that all three pair interactions are between the fragment pairs connected by hydrogen bonds (Figure 3b), clarifying the physical nature of binding. All interactions larger than 1 kcal/mol are shown. All of them are attractive (the largest repulsive interaction, not shown, is 0.4 kcal/mol).

As found in simulations, the interactions for hydrogen bonds between OH groups of monosaccharides and phosphate groups in nucleotides stand out with large values of −23.7, −26.5, and −38.1 kcal/mol (Figure 3c). These values correspond to fragment–fragment interactions and include interactions other than hydrogen bonding; in this case, charge-dipole interactions are the main factor for T1 and C1. Ar9-T1 is the largest because it involves two hydrogen bonds (Figure 3a). It is interesting and perhaps less intuitive that neutral A1 has a large interaction. To the terminal –PO_3_^2−^ group two sodium ions are added as counterions so that the total charge is zero. However, in the optimized structure, one sodium atom is separated far away from AG. Therefore, the Coulomb field from it is weaker, and effectively the interaction is increased due to the attraction to the −2 charge on the phosphate only partially compensated by the counterions. The second strand, with T2, A2, G2, and C2, does not interact with AG. The main binder in the aptamer in the first strand is T1, and A1 and C1 are also strongly bound. G1 is geometrically separated and is not bound to AG. These results pinpoint which nucleotides bind to AG, and provide hints as to how to modify the aptamer to strengthen or weaken the binding.

### 2.4. Effects of AS-FeAG on Carcinoma Cells in Vitro

**Figure 4:**
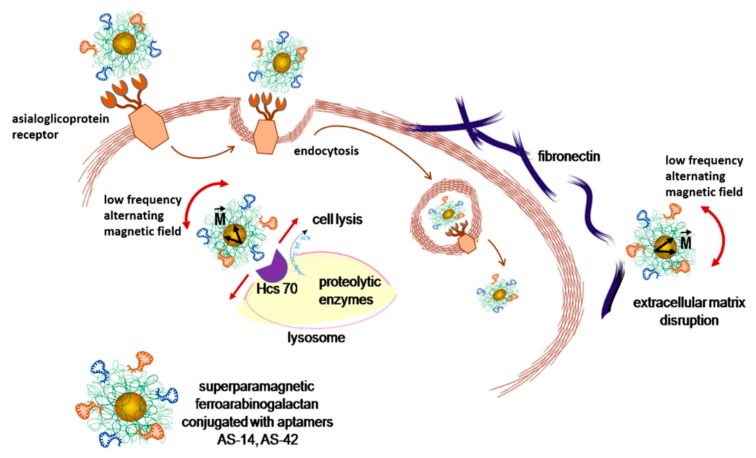
Schematic representation of asialoglycoprotein receptor-mediated delivery and cell damage by AS-FrFeAG nanoparticles at a low alternating magnetic field. Due to a magnetic moment (M) of nanoparticles, they oscillate in an alternating magnetic field. AS-FrFeAG binds to fibronectin via aptamer AS-14 and disrupts extracellular matrix; AS-FrFeAG is internalized via asialoglycoprotein receptors and binds to Hsc70 via aptamer AS-42 causing lysosome damage and leakage of proteolytic enzymes resulting in cell lysis.

Coating with AG enhances biocompatibility and promotes nanoparticle cell internalization via asialoglycoprotein receptors [32]. In the absence of a magnetic field, the orientation of magnetic domains of superparamagnetic particles is random. An external magnetic field causes the reorientation of magnetic domains along the field. Therefore, a low frequency (50 Hz) alternating magnetic field (LFAMF) rotates superparamagnetic particles of AS-FeAG clockwise back and forward. Binding the particles through aptamer AS-14 with fibronectin and following LFAMF treatment disrupts cellular adhesion and disintegrates cancer cells (Figure 4). Hsc70, a target of AS-42, is localized in the cytoplasm and lysosomes [34,35]; thus, the damage of lysosome integrity by nanoparticles rotating in LFAMF could cause cell lysis from inside.

**Figure 5:**
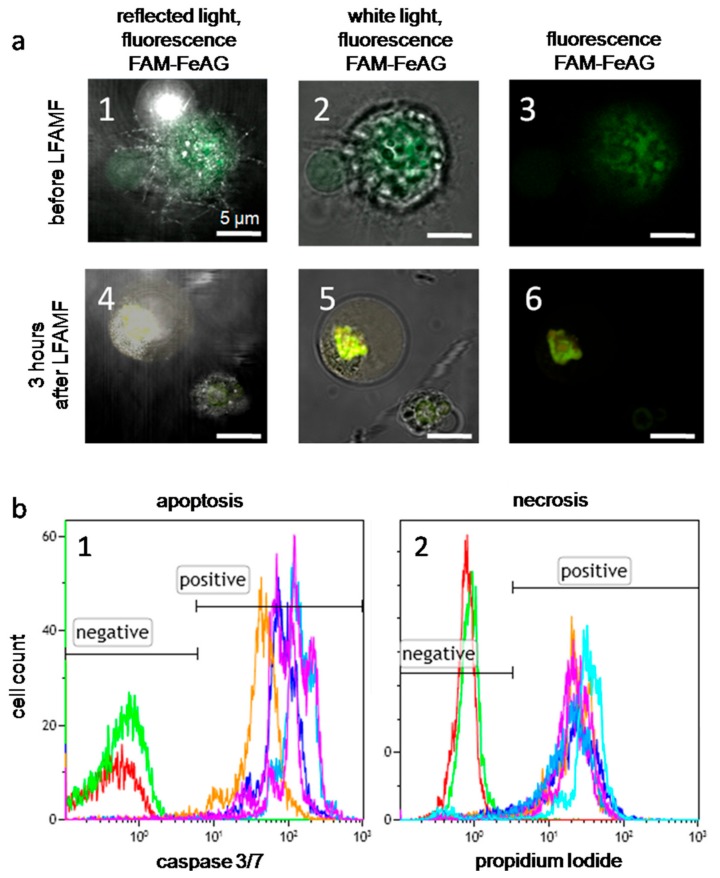
Effects of AS-FeAG and low frequency alternating magnetic field (LFAMF) in vitro. (**a**) Fluorescence microscopy of Ehrlich cells with FAM-labeled As-FeAG before and after LFAMF treatment for 10 min represented in reflected light with fluorescence (**a1**,**4**), white light with fluorescence (**a2**,**5**) and only fluorescence (**a3**,**6**). Scale bar: 5 µm. (**b1**) Flow cytometry analysis of apoptosis 3 h after the treatment using the caspase 3/7 activity; (**b2**) the level of necrosis determined by propidium iodide accumulation in dead cells. The red curve corresponds to intact cells without staining; the green curve represents the basic level of non-treated cells; purple—to cells treated with FrFeAG (6%); blue—to FrFeAG (4.5%), dark blue—to FeAG (6%), orange—to FeAG (4.7%).

In laser scanning microscopy binding analyses of FeAG nanoparticles to Ehrlich carcinoma cells, aptamers (AS-14 and AS-42) were labeled with a FAM label and attached to FeAG. AS-42 modified FeAG nanoparticles entered a cell and were concentrated around the nucleus; AS-14 FeAGs bound to the cell membrane and stained the cell surface (Figure 5(a1–3)). Three hours after the treatment with the alternating magnetic field, the particles on the cell membranes were detached from the cell, and the particles inside the cells formed agglomerates (Figure 5(a4–6)).

Different fragmentation of AG demonstrated a different effect on cell viability in vitro. Apoptosis (Figure 5(b1)) and necrosis (Figure 5(b2)) of Ehrlich cells with As-FeAG before and after LFAMF treatment for 10 min were determined. FeAG from the fragmented AG showed higher efficacy than non-fragmented AG, probably, because of a thinner layer of AG. Moreover, viability slightly depends on the Fe content; AS-FrFeAG with 6% of Fe was the most effective and was chosen for in vivo experiments.

### 2.5. Magnetic Resonance Imaging of Carcinoma with AS-FeAG In Vivo

**Figure 6:**
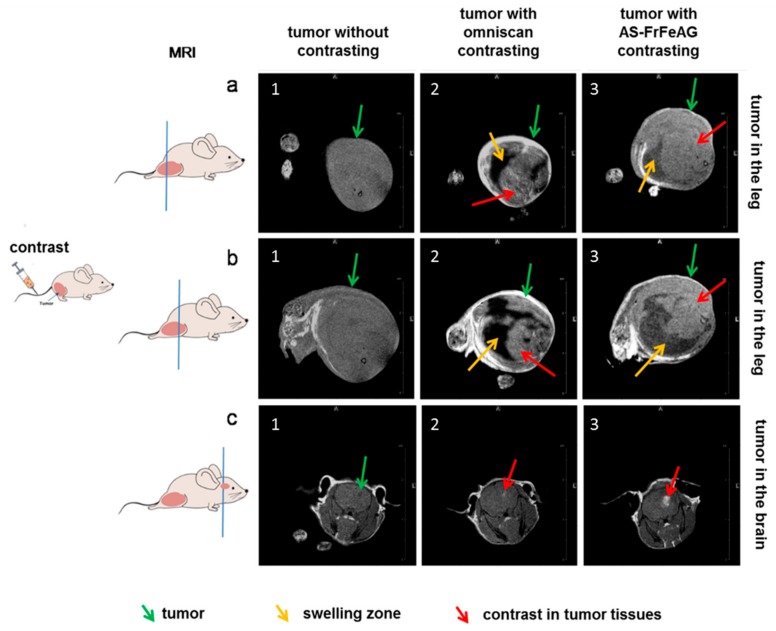
Application of AS-FrFeAG as a contrast agent for tumors in magnetic resonance imaging (MRI). A mice with solid Ehrlich carcinoma transplanted into the right leg (**a**,**b**) and brain (**c**) without contrasting (**1**); with omniscan (**2**), and AS-FrFeAG (**3**) as a contrast agent.

Solid Ehrlich carcinoma transplanted into the right leg and brain of mice were visualized in MRI by the aptamer conjugated FrFeAG (6%). It was evaluated at 30 min after intravenous injection of AS-FrFeAG (8 μg/kg) or immediately after Omniscan (a gadolinium-based contrast agent) (1.9 μM/kg) (3 animals in each group). Mice were sedated with thiopental and scanned. The experiment showed that AS-FrFeAG was accumulated in the tumors in a leg similar to gadolinium (Figure 6(a1–3,b1–3), making it more contrasting (Figure 6(a3,b3)), but for some reason with lower resolution than gadolinium (Figure 6(a2,b2)). In addition, AS-FrFeAG penetrated the blood-brain barrier and concentrated in the tumor in the brain (Figure 6(c3)) similarly to gadolinium (Figure 6(c2), while without the contrast the tumor is almost not visible (Figure 6(c1)).Potentially, AS-FrFeAG could be used as a contrast agent for tumors in the brain.

**Figure 7:**
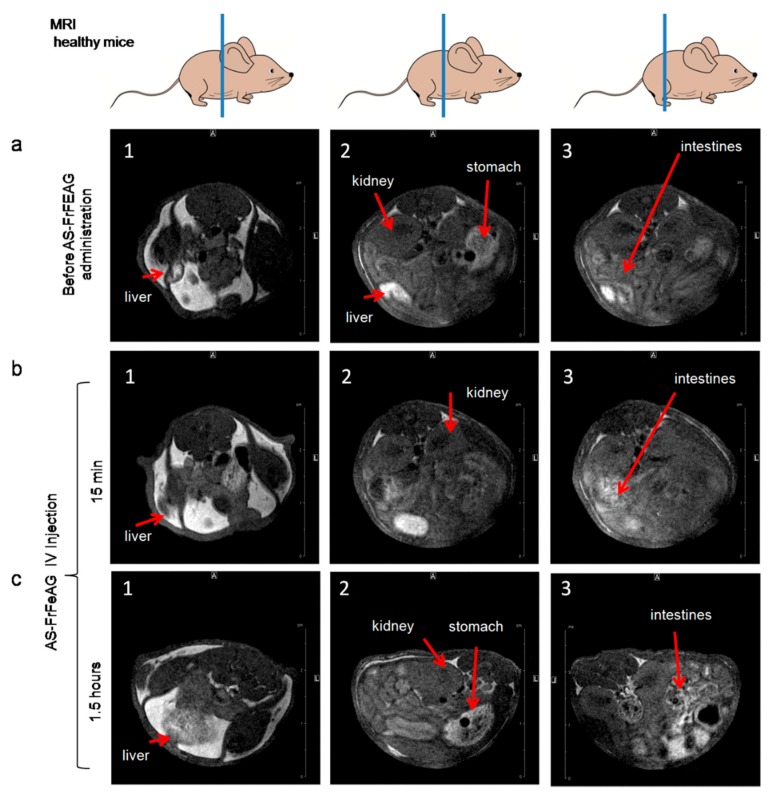
Distribution of AS-FrFeAG in different organs of mice analyzed by MRI before (**a**) and 15 min (**b**), 1.5 h (**c**) after the intravenous administration.

We made an attempt to demonstrate the organ distribution of As-FrFeAG in healthy mice after intravenous injection (Figure 7). It is known that AG can be used as a hepatic drug delivery agent; this polysaccharide is accumulated in liver cells due to asialoglycoprotein receptors [36]. In MRI experiments, we did not see AS-FrFeAG accumulation in the liver after 15 min and 1.5 h of intravenous (IV) administration. The liver looked very bright already on the MRI image before As-FrFeAG injections. That is why we did not see the contrast from AS-FrFeAG in the liver. AS-FrFeAG was detected in the intestines 1.5 h after injection, where it might have come from the liver.

### 2.6. Effects of AS-FeAG on Carcinoma In Vivo

The therapeutic potency of ferroarabinogalactan in LFAMF and its efficacy in the case of targeted delivery by the aptamers were estimated using the same tumor model. Briefly, AS-FrFeAG (0.8 μg/kg), FrFeAG (1.6 μg/kg), an aptamer pool of AS-14 and AS-42 (0.4 mg/kg) were injected intravenously to mice (5 animals in each group). Then, 30 min after injection, animals were placed into LFAMF for 10 min. The treatment procedures were repeated three times. Non-treated animals were used as control. The treatment caused inflammation and tumor degradation (Figure S8). Histological changes in cancer tissues allowed us to conclude that targeting with the aptamers enhanced the therapeutic effects of AS-FeAG nanoparticles in LFAMF.

**Figure 8:**
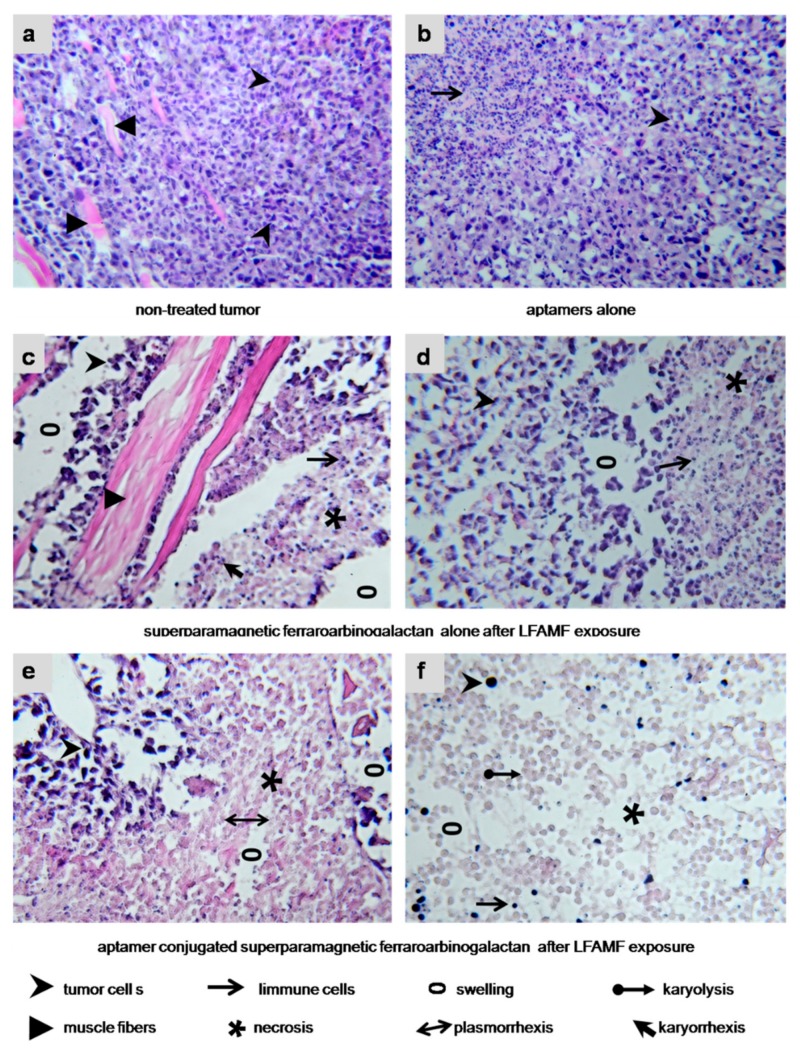
Histological features of the treated tumors. (**a**) Non-treated Ehrlich carcinoma. The invasive tumor has a solid structure composed of atypical cells with pleomorphic, hyperchromatic nuclei of different shapes and volume and grows into the muscle tissue. No immune response. (**b**) Treated with an aptamer mix. Carcinoma cells are with cytoplasm vacuolization; lymphocytic infiltration is moderate. (**c**,**d**) FrFeAG and LFAMF treated carcinoma has scattered tumor necrosis among the remaining carcinoma tissue with inflammatory infiltration around them. (**e**,**f**) AS-FrFeAG in LFAMF caused irreversible damaging effects on the treated tumor, such as karyo and plasmolysis, and karyo and plasmorrhexis. Large tumor necrosis area, on the periphery of which remains small amounts of dead carcinoma cells with destructive changes of the cancer tissue microenvironment. Visible inflammatory infiltration of segmented leukocytes and swellings observed. Magnification × 100.

Carcinoma cells in a control group looked viable; a growing tumor destroyed muscle tissue; no immune response was registered (Figure 8a). The control treatment with the aptamer pool in DPBS and LFAMF caused inflammatory infiltration with segmented leucocytes (Figure 8b). The treatment with FrFeAGcaused partial necrosis of tumor tissues (mostly around the blood vessels), disruption of cell adhesion, inflammatory infiltration with segmented leucocytes, and swelling (Figure 8c,d). These results showed that the treatment with AS-FrFeAG nanoparticles in LFAMF caused tumor destruction and local inflammatory response.

Despite the dose of AS-FrFeAG being reduced two times compared with FrFeAG, the therapeutic effect was significant (Figure 8e,f). Total tumor necrosis was observed; remaining small amounts of carcinoma cells had irreversible changes: karyorrhexis, karyolysis, plasmorrhexis. This treatment caused immune infiltration of segmented leukocytes, swelling, and destructive changes of tumor tissue extracellular matrix (Figure 8e). Large areas of lysed cells without nucleolus were found with remaining sporadic cells and lymphocytes (Figure 8f).

## 3. Methods

### 3.1. Production of Superparamagnetic FeAG

Superparamagnetic FeAGs were obtained from an industrial sample of AG (molecular weight 16,645 Da) and fragmented AG (molecular weight 8380 Da) of Siberian larch (INPF “Chemistry of Wood”, Irkutsk, Russia) and additionally purified by reprecipitation from water to ethanol. Fragmentation was performed using the method [37]. Iron-based FeAGs from AG or fragmented AG were synthesized according to the procedure described in the patent [38]. Detailed characterization is represented in Supplementary Materials (Table S1, Figure S2–7).

The schematic representation of the synthesis superparamagnetic FeAG is presented in Figure 1a. AG in deionized water was mixed with an aqueous solution of salts of FeSO_4_ × 7H_2_O and FeCl_3_ × 6H_2_O in a 1:2 ratio and held at room temperature for 30 min; afterward, a 25% solution of ammonium hydroxide was added, heated in a water bath for 15 min, and cooled. The resulting products were filtered and purified by triple precipitation from the aqueous solution to ethyl alcohol. Superparamagnetic FeAG was synthesized from a full length (or fragmented) AG with a different Fe content. The amount of the mixture of Fe salts taken for the synthesis was 0.76 and 0.96 mmol. An electron microscope (Hitachi TM3000, Tokyo, Japan) was used to estimate the percentage ratio of Fe. EM spectra were processed with the software Quantax 70 (Bruker) for Hitachi TM3000 (Table 1).

### 3.2. Conjugating FeAG with Aptamers

The aptamer mix (1:1) from AS-14 (5′-CTC CTC TGA CTG TAA CCA CGA AGG TGT CGG CCT TAG TAA GGC TAC AGC CAA GGG AAC GTA GCA TAG GTA GTC CAG AAG CC-3′), AS-42 (5′-CTC CTC TGA CTG TAA CCA CGT CAA TGG GTG ATA TAT GCA GGT TAC GCT GGC TAG TTG AAA GCA TAG GTA GTC CAG AAG CC-3′) in concentration of 500 nM in DPBS buffer containing 0.9 mM CaCl_2_ and 0.49 mM MgCl_2_, was mixed 1:1 with FeAG (800 µg/mL in DPBS) incubated at room temperature for 30 min. Ca^2+^ and Mg^2+^ are essential for the correct formation and maintenance of the aptamer tertiary structure, important for further experiments.

### 3.3. Molecular Modeling of AG Interaction with an Aptamer

Based on the nanoparticle shown in the furthest right part of Figure 1a, a suitable model for simulations was chosen, which excludes the ferrocluster and includes a representative part of an arabinogalactan (5 sugar units) and eight nucleotides from a DNA aptamer (double-stranded A-T, T-A, C-G, G-C pairs at the two terminal sections of the aptamer), see Figure 1a,b. To make the simulations more realistic, counterions (sodium cations) were added around each negatively charged phosphate group in the aptamer (1 counterion to =PO_2_^−^ and two counterions to terminal –PO_3_^2−^ groups), except that two phosphate groups of T1 and C1 (Figure 1b) were not given a counterion, because these groups were assumed to bind directly to arabinogalactan. The numbering of nucleotides includes a digit 1 or 2 depending on which strand they belong to; the strand 1 is the closest to the aptamer.

The molecular structure was optimized (energy-minimized with the threshold of 10^−4^ hartree/bohr) using the fragment molecular orbital method (FMO) [39] at the level of the two-body FMO expansion for third-order density-functional tight-binding (FMO2-DFTB3) using 3ob parameters [40], combined with the conductor polarizable continuum model of solvation (C-PCM) [41]. FMO-DFTB/PCM is a fast and accurate method, as was demonstrated in comparing optimized protein structures with experiments [41]. The fragmentation was done according to the chemical nature (see Figure 4), by dividing arabinogalactan and aptamer into 5 and 8 fragments, respectively. Sodium atoms were assigned to the nearby nucleotide fragment. T1 and C1 fragments have a −1 charge, and all other fragments are neutral.

To reveal the physical picture of the binding between arabinogalactan and an aptamer, pair interaction energy decomposition analysis (PIEDA) [42] was employed at the level of second-order Møller–Plesset perturbation theory (MP2) with the 6-31G(d,p) basis set and C-PCM. All FMO calculations were performed using [43]. MP2 is a good method for describing both electrostatic and non-electrostatic (hydrophobic) interactions at the ab initio quantum-mechanical (QM) level without using any parameters. Due to its QM nature, it describes polarization and charge transfer as well. The solvent screening, effectively shielding the strong ionic interactions, was taken into account [42].

Although PIEDA provides many components [44], in this work, for simplicity, some of them were added together, so that the fragment–fragment interaction is divided into electrostatic (ES), quantum-mechanical (QM) and van-der-Waals (vdW) components. ES includes solute–solute interaction between polarized electron densities of the two fragments, nuclear terms, and solute-solvent screening. QM includes charge transfer and exchange-repulsion (short-range repulsion between electrons). vdW is computed from the electron correlation energy in MP2 and mainly describes hydrophobic interactions.

### 3.4. Flow Cytometry Analyses of Cells Treated with AS-FeAG in LFAMF

Mouse Ehrlich ascites cells were cultured in 35 × 10 mm cell culture dishes (CELLSTAR^®^, Frickenhausen, Germany) in Dulbecco’s modified Eagle’s medium (DMEM; Sigma-Aldrich, St. Louis, MO, USA). The medium was additionally supplemented with 100 U/mL penicillin, 100 U/mL streptomycin, and 5% (*v/v*) fetal bovine serum (FBS). All cultivation operations were performed in a humidified atmosphere containing 5% CO_2_ at 37 °C.

One million cells in 500 mL of colorless high glucose DMEM medium with calcium and magnesium were incubated with different compositions of FeAG at 1:1000 ratios (final concentration 1 × 10^9^ particles per 1 mL) or only DPBS for 30 min at 37 °C in a humidified atmosphere containing 5% CO_2_. All samples were prepared in triplicate. After incubation, the cells were kept in a magnet producing LFAMF for 10 min.

The level of apoptosis was estimated by caspase 3/7 activity 3 h after the treatment using CellEvent™ Caspase-3/7 Green Detection Reagent (Molecular Probes, ThermoFisher Scientific, Austin, TX, USA) according to manufactures protocol using a flow cytometer (FC-500, Beckman Coulter, Irving, TX, USA). The level of necrosis was determined by propidium iodide (Sigma Aldrich, St. Louis, MO, USA) accumulation in dead cells 5 h after the treatment in accordance with the manufacturer’s protocol.

### 3.5. Generation of LowFrequency Magnetic Field

Helmholtz coils were used to generate an alternating low-frequency magnetic field; the equipment was specially designed for these experiments (Figure S1). The copper wire (0.53 mm in diameter) was wounded on two cylinders with an inner diameter of 20 cm; the external diameter of the coil was 22 cm, and the resistance of the coil was 21.4 Ω. Parameters of the magnetic field were the following: sinusoidal waveform, 50 Hz frequency, and 100 Oe intensity. The coils ensure the creation of a uniform magnetic field in a volume of 15 × 15 × 15 cm. The magnetic field strength of 100 Oe was controlled by a TL-5 millitesla-meter. Up to 5 mice could be simultaneously placed in the working area of the rings. The coil dissipated approximately 1.7 W of power. The temperature of the coil, the samples, and the animals during the procedure were controlled during the treatment. After each treatment, the coil was switched off for 10 min in order to avoid heating.

### 3.6. Ethics Committee Approval

The animal protocol was approved by the Krasnoyarsk State Medical University Local Committee on the Ethics of Animal Experiments (ethical code: #77; June 26, 2017). This study was carried out according to the recommendations in the National Institute of Health’s Guide for the Care and Use of Laboratory Animals. All procedures were performed under anesthesia, and all efforts were made to minimize the suffering of the animals.

### 3.7. Mice Tumor Models

White Imprinting Control Region (ICR) mice were taken for the experiments. All mice were six weeks old and weighed approximately 25 g.

#### 3.7.1. Solid Tumor in the Leg

Three million Ehrlich ascites carcinoma cells in 100 µL of DPBS were transplanted subcutaneously into the right leg. The first treatment procedure was performed when the tumor became visible on day four after the tumor transplantation; the following procedures were on days 7 and 10. MRI studies were performed on day nine after tumor transplantation when the approximate diameter of the leg with the tumor was 1.5 cm.

#### 3.7.2. Solid Tumor in the Brain

Mice heads were shaved with shaving cream, and 70% alcohol was used to sterilize the skin. Animals were anesthetized by isoflurane inhalation, and a 0.7-cm midline scalp incision was made. A hole for injection was made by slow, gentle rotation of a 0.33-mm insulin syringe in front of the coronal suture, 3 mm to the right of the midline. Then, 6 µL of Ehrlich ascites carcinoma cell suspension (100 thousand cells in DPBS per mice) was injected using a 50 µL Hamilton syringe 1705LT with a disposable needle, through the hole. A silicone tube was placed on the needle permitting an injection depth of 2 mm from the bone. The skin was closed and sawed with the surgical needle. The whole procedure was performed in a sterile environment with no use of antibiotics. MRI studies were performed on day three after tumor transplantation; the approximate diameter of the tumor according to histological data was 1–2 mm.

### 3.8. In Vivo Treatments with AS-FeAG in LFAMF

The animals in the experimental group were treated using aptamer conjugated superparamagnetic FrFeAG (6%) followed by placing in the low frequency alternating magnetic field. The control groups were the following: untreated mice, treatments with an aptamer mix, or FrFeAG alone.

Every third day, starting from day four after tumor transplantation until day ten (on days 4, 8, and 10 after the tumor transplantation, three times total), animals were placed in LFAMF for 10 min, 30 min prior to this procedure the mice were administered by tail vein injections as follows:

Group 1: Untreated control;
Group 2: Injection of the aptamer mix of AS-14 and AS-42 in 100 μL DPBS (400 μg/kg);
Group 3: Injection of FrFeAG (6%) in 100 μL DPBS (1.6 μg/kg);
Group 4: Injection of the aptamer mix (AS-14, AS-42) conjugated with FrFeAG (6%) in 100 μL DPBS (0.8 μg/kg);

After 30 min, animals were placed inside the magnet and were treated with a low frequency alternating magnetic field for 10 min.

### 3.9. Magnetic Resonance Imaging

MRI visualization was carried out using Avance DPX 200 spectrometer (Bruker BioSpin GmbH, Rheinstetten, Germany) equipped with imaging accessories in the following configuration: a superconducting magnet with an 89 mm diameter vertical bore, a water-cooled and self-shielded Bruker GREAT 3/60 gradient unit, a probe PH MINI 0.75 (maximum gradient strength up to 292 mT/m), and a 38 mm internal diameter birdcage coil tuned and matched to 1H nuclear resonance frequency of 200.13 MHz. Slice selective 2D NMR images were acquired using the multi-slice multi-echo (MSME) technique supplied by Paravision 4.0 software (Bruker BioSpinGmbH, Ettlingen, Germany). The parameters of image acquisition were as follows: slice thickness of 0.71 mm; a field of view (FOV) of 40 mm; matrices of 256 × 256 pixels, which provided the spatial resolution of 156 μm per pixel within the slice. The repetition time (TR) and echo time (TE) were adjusted to 600 ms and 4.7 ms, respectively, to provide the T_1_-weighting on the images.

The time of image acquisition (TA) was 10 min. It resulted from the TR and TE parameters and the number of scans.

### 3.10. Histological Analysis of Tumor Tissues

Tumors were harvested on day 12 after transplantation (2 days after the last procedure) and placed in a 3.7% formalin solution. A series of 10 μm tissue sections were prepared using cryostat HM 525 (Carl Zeiss, Germany). These sections were fixed on glass slides and stained with hematoxylin and eosin. Evaluation of histological changes of the tumor tissue sections after therapy was performed on a microscope (Axioskop 40, Carl Zeiss, Germany).

## 4. Conclusions

Conventional anti-cancer drugs are highly toxic, and sometimes of low efficacy and even may increase tumor malignancy. In recent years, novel nanotechnologies involving physical methods of tumor destruction with nanoparticles have been developed. Despite these techniques being highly effective, they also have limitations such as cell toxicity and low selectivity due to the accumulation of nanoparticles in non-targeted tissues and organs.

In the current work, we made an attempt to improve and optimize magnetodynamic therapy with nanoparticles. In order to increase biocompatibility, superparamagnetic iron particles were synthesized in the polymer matrix of AG. AG of Siberian larch is well known for its antioxidant, antimicrobial, anti-mutating, and immunostimulating properties, and can provide high biocompatibility for magnetic particles [45]. AG is internalized into a cell via asialoglycoprotein receptors (ASGPR) and, therefore, can be used for the delivery of small or large molecules and particles inside ASGPR-positive cells. Here we coated 10 nm superparamagnetic iron nanoparticles with AG. The coating with the fragmented AG increased the therapeutic effect due to particle size reduction. It is important to note that liver cells overexpress ASGPR [46]. Therefore, FeAG will be mostly accumulated in liver parenchymal cells. Conjugating FeAG with anticancer DNA aptamers allowed us to reduce the dose of the particles and to increase FeAG uptake by cancer cells. The aptamers enhanced the accumulation of the nanoparticles near cancer cells with following damage of the cells by a magnetic field. LFAMF did not cause local hyperthermia and, therefore, did not affect healthy organs and surrounding tissues. In addition, an excess of AG promoted the excretion of tumor decay products due to its antioxidant, immunomodulating, and detoxification properties. Targeted magnetodynamic nanotherapy with aptamer-conjugated superparamagnetic nanoparticles opens up a new venue for low toxic, minimally invasive, and directed theranostics of cancers.

## Figures and Tables

**Table 1:** Different compositions of ferroarabinogalactans (FeAGs).

Sample	Molecular Mass of the Starting AG, Da	Amount of IronSalts, Mmol	Product Yield, %	Fe Content, %
FeAG (4.7%)	16,645	0.75	81	4.7
FrFeAG (4.5%)	8380	0.75	82	4.5
FeAG (6%)	16,645	0.96	80	6.0
FrFeAG (6%)	8380	0.96	75	6.0

## Supplementary Materials

The following are available online at https://www.mdpi.com/2072-6694/12/1/216/s1. Figure S1: The equipment of Helmholtz coils, Figure S2: HPLC chromatograms, Figure S3. A 13C NMR spectrum of AG (sample AG-1), Figure S4. Particle sizes of iron-containing AG (MM 8380 Da), Figure S5. An IR spectrum of iron-containing AG (MM 8380 Da), Figure S6. TEM images of iron-containing AG (MM 8380 Da), Figure S7. Microelectron diffraction of iron-containing AG (MM 8380 Da), Figure S8: Treatments with FrFeAG and AS-FrFeAG in a low alternating magnetic field caused inflammation, abscesses and ulceration comparing with the control without any treatment, Table S1: Characterization of arabinogalactan samples.
