# Supplementary material for: Aptamer-Conjugated Superparamagnetic Ferroarabinogalactan Nanoparticles for Targeted Magnetodynamic Therapy of Cancer
Source: Cancers (Basel). 2020 Jan 15;12(1):216. doi: 10.3390/cancers12010216 (PMC7017168; doi:10.3390/cancers12010216)
Supplement: Supplementary file 1 [file cancers-12-00216-s001.pdf]

Supplementary Information: Aptamer-Conjugated Superparamagnetic Ferroarabinogalactan Nanoparticles for Targeted Magnetodynamic Therapy of Cancer

Olga S.Kolovskaya, Tatiana N. Zamay, Galina S. Zamay, Vasily A. Babkin, Elena N. Medvedeva, Nadezhda A. Neverova, Andrey K. Kirichenko, Sergey S. Zamay, Ivan N. Lapin, Evgeny V. Morozov, Alexey E. Sokolov, Andrey A. Narodov, Dmitri G. Fedorov, Felix N. Tomilin, Vladimir N. Zabluda, Yulia Alekhina, Irina V. Garanzha, Kirill A. Lukyanenko, Yury E.Glazyrin, Valery A. Svetlichnyi, Maxim V. Berezovski and Anna S. Kichkailo

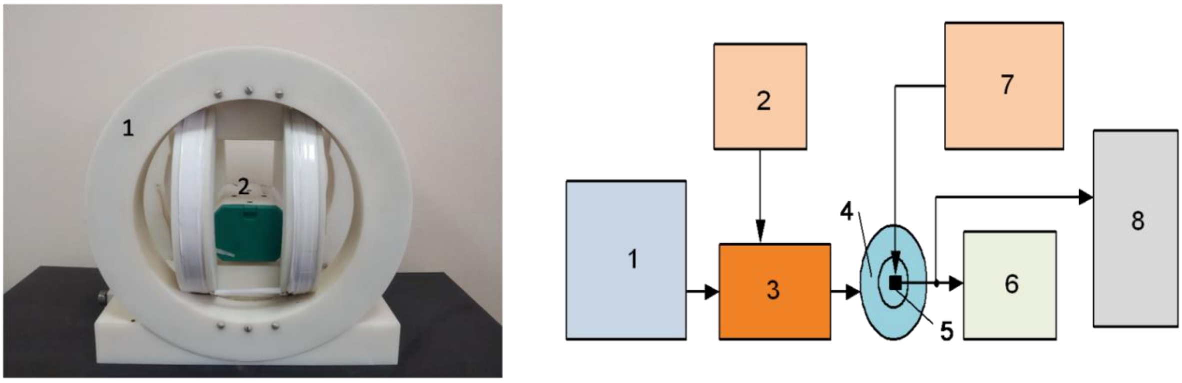

Figure S1. (**A**) Helmholtz coils for generation of a low frequency magnetic field (1); a box for an experimental mouse (2). (**B**) A scheme for installation of optimal magnetodynamic therapy. (1) – a signal generator, (2) – a stabilized DC voltage source, (3) - a bridge amplifier, (4) - Helmholtz coils, (5) – a Hall sensor, (6) - a microvoltmeter, (7) – a 5 V power supply for a Hall sensor, (8) - an oscilloscope.

Arabinogalactan characterization

An industrial sample of arabinogalactan (AG) was obtained using a patent (No. 2413432 (RF), Babkin D.V., Ugreninov A.A. Method for producing arabinogalactan, TU 9354-041-64843061-13) and provided by INPF Wood Chemistry LLC (Irkutsk) (Table S1, sample AG-1), which was further purified by precipitation from water into ethanol (Table S1, sample AG-2) and fragmented according to the method [1] (Table S1, sample AG-3).

Table S1. Characterization of arabinogalactan samples.

Sample	AG content, % from the powder	The content of phenolic impurities in terms of dihydroquercetin, %	Moisture, %	Molecular weiht, Da	M_w/M_n
AG-1	99.4	0.55	5.5	16645	1.28
AG-2	100	-	2.0	16645	1.27
AG-3	100	-	1.9	8380	1.37

The average molecular weights of arabinogalactan samples were determined by HPLC using an Agilent 1260 chromatographic system on a PL aquagel-OH-40 column 8 μm, 300 × 7.5 mm, with a PL aquagel-OH Guard column 8 μm, 50 × 7.5 mm, calibrated using dextran with molecular weights of 25, 12, 5 kDa and D-galactose. Column temperature was 25 °C. Spectra were recorded on a refractometric detector at 30 °C. The eluent was 0.1 M $LiNO_3$ with a feed rate of 1.0 mL/min and loop volume of 20 μL.

According to HPLC results, all samples had a single peak and narrow molecular weight distribution (Table S1, figure S1).

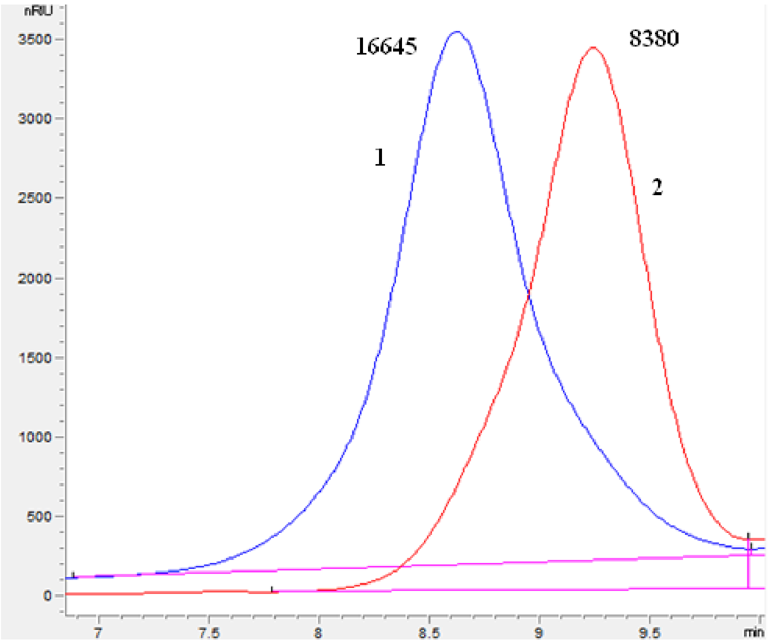

Figure S2. HPLC chromatograms: 1 - purified AG (sample AG-2); 2—fragmented AG (sample AG-3). 13C NMR spectra of the samples were recorded on Bruker DPX 400 and AV 400 spectrometers with an operating frequency of 100.61 MHz at 298K (solvent—D_2O). IR spectra were recorded in KBr tablets on a Varian 3100 FT-IR spectrophotometer in the range 400-4000 cm^{-1}.

The absence of absorption in the region of 1700 cm^{-1} in the IR spectra, as well as signals in the region of 200 - 220 ppm in 13C NMR spectra indicates the absence of acidic carbonyl groups in AG macromolecules (Figure S3).

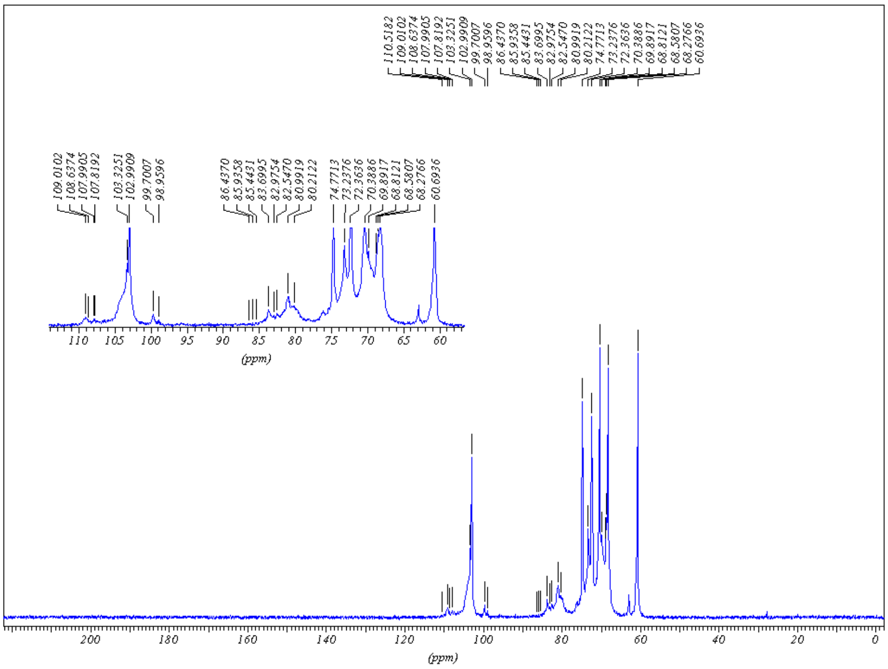

Figure S3. A 13C NMR spectrum of AG (sample AG-1).

Ferroarabinogalactan nanoparticles characterization

X-ray energy dispersive microanalysis determined the sizes of individual particles of iron-containing arabinogalactan varying in the range of 1-3.5 μm, the average size of which was 1.96 μm (Figure S4).

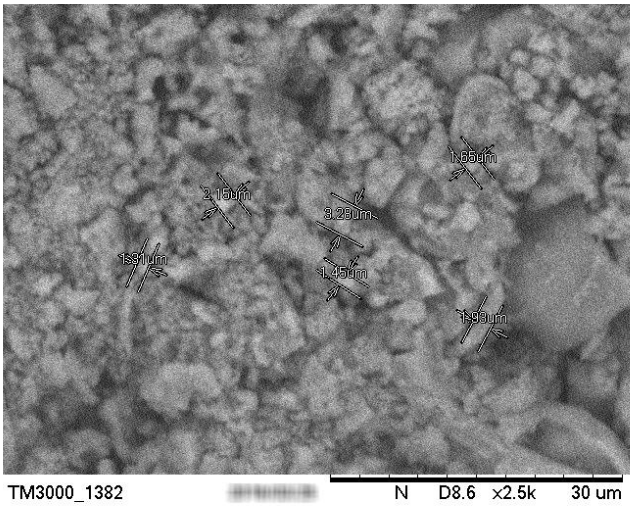

Figure S4. Particle sizes of iron-containing AG (MM 8380 Da).

Figure S5 shows the IR spectrum of an iron-containing AG with a molecular weight of 8380 Da, which is identical to the IR spectra of the initial samples of arabinogalactan.

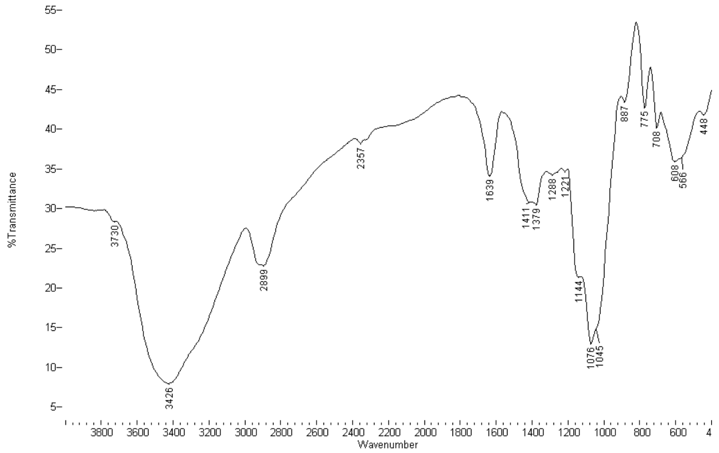

Figure S5. An IR spectrum of iron-containing AG (MM 8380 Da).

In order to identify the sizes of magnetic particles ferroarabinogalactan samples have been analysed using Transmission electron microscopy (TEM) (Figure S6) Dark spots are iron oxides, surrounded by arabinogalactan. The size of the nanoparticles is about 5 nm.

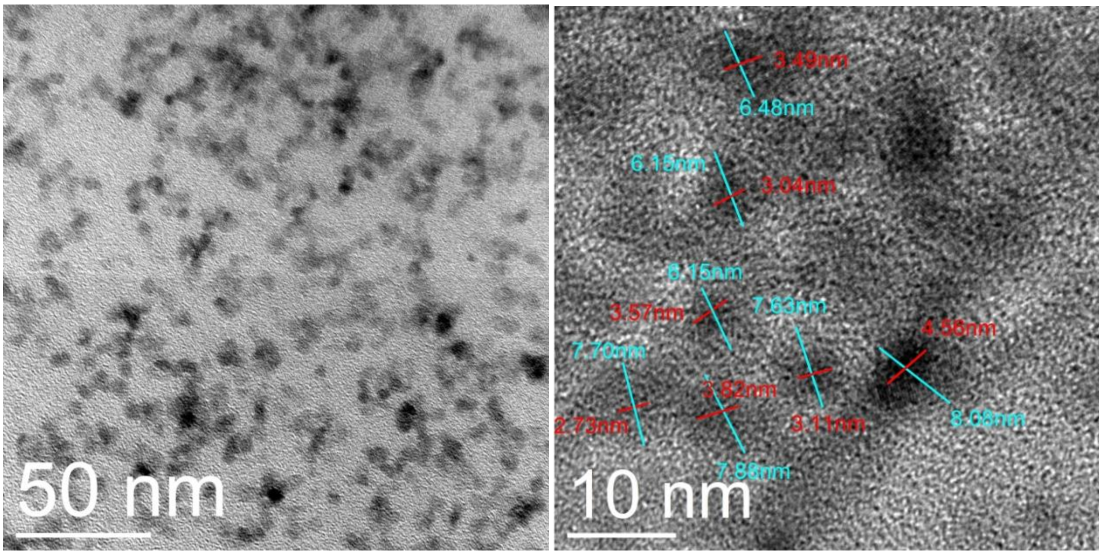

Figure S6. TEM images of iron-containing AG (MM 8380 Da).

In addition, microelectron diffraction analysis was performed. According to the deviation radius, the particles in ferroarabinogalactan were made from magnetite.

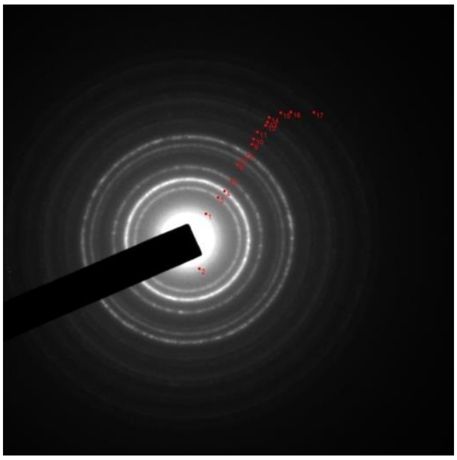

Figure S7. Microelectron diffraction of iron-containing AG (MM 8380 Da).

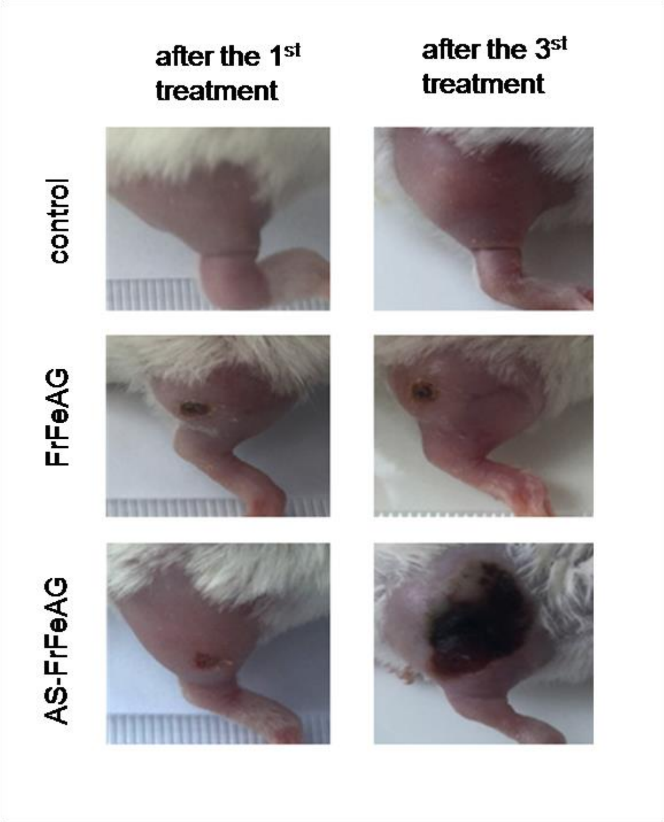

Figure S8. Treatments with FrFeAG and AS-FrFeAG in a low alternating magnetic field caused inflammation, abscesses and ulceration comparing with the control without any treatment.

References

1. Ponder G.R., G.N. R. Arabinogalactan from Western Larch, Part II; A Reversible Order-Disorder Transition. *Journ. Carbohydr. Chem.* **1997**, *16*, 195–211.

© 2020 by the authors. Licensee MDPI, Basel, Switzerland. This article is an open access article distributed under the terms and conditions of the Creative Commons Attribution (CC BY) license (http://creativecommons.org/licenses/by/4.0/).
